# Nanoparticles-Assisted Stem Cell Therapy for Ischemic Heart Disease

**DOI:** 10.1155/2016/1384658

**Published:** 2015-12-29

**Authors:** Kai Zhu, Jun Li, Yulin Wang, Hao Lai, Chunsheng Wang

**Affiliations:** ^1^Department of Cardiac Surgery, Zhongshan Hospital, Fudan University, Shanghai 200032, China; ^2^Shanghai Institute of Cardiovascular Disease, Shanghai 200032, China

## Abstract

Stem cell therapy has attracted increasing attention as a promising treatment strategy for cardiac repair in ischemic heart disease. Nanoparticles (NPs), with their superior physical and chemical properties, have been widely utilized to assist stem cell therapy. With the help of NPs, stem cells can be genetically engineered for enhanced paracrine profile. To further understand the fate and behaviors of stem cells in ischemic myocardium, imaging NPs can label stem cells and be tracked *in vivo* under multiple modalities. Besides that, NPs can also be used to enhance stem cell retention in myocardium. These facts have raised efforts on the development of more intelligent and multifunctional NPs for cellular application. Herein, an overview of the applications of NPs-assisted stem cell therapy is given. Key issues and future prospects are also critically addressed.

## 1. Introduction

Ischemic heart disease and its fatal sequelae are among the main causes of death worldwide [1, 2]. Over the past half-century, conventional treatments, including medicine and surgery, have yielded dramatic decline in mortality. Despite the enormous advances, these treatments merely lead to the temporary delay in ischemia progression. Heart transplantation could be the only definite and long-term therapy but is seriously limited by the deficiency of organ sources and inevitable immunological rejection [3–5]. In the last decade, stem cell transplantation has emerged as a potential approach to repair the ischemic myocardium. In this context, a wide variety of stem cells have been considered as potential candidates for cardiac repair. Some of them, such as bone marrow-derived stem cells, have been translated into early phase clinical trials [6]. However, therapeutic effect and evaluation of stem cells need further optimization in the near future.

Nanotechnology has been considered as a great breakthrough in this century. This technology, through controlling materials at nanoscale, has driven revolutionary developments in almost all fields. Nanoparticles (NPs), whose diameter ranges from 1 to 100 nm, have been widely used for fast-diagnosis, molecule delivery, and tissue engineering, which has been situated at the frontier in biomedical research. Their unprecedented advance has paved the way for assisting stem cells therapy [7]. Here, we reviewed the current knowledge and future prospects for NPs-assisted stem cell therapy for cardiac repair in ischemic heart disease.

## 2. Biosafety Risks of NPs

Before NPs can be translated into clinic, biosafety is one of the most important concerns. The intrinsic nanofactor of NPs can cause unexpected cytotoxic risks [8]. Due to their nanoscaled sizes, NPs can easily transport across cell membrane and reach the crucial organelles, including endoplasmic reticulum, mitochondria, and nucleus. And high surface area over volume ratio augments their interaction with cellular components [9]. As foreign materials to cells, NPs may affect cell homeostasis through several mechanisms. Firstly, the large reaction surfaces of NPs yield massive reactive oxygen species (ROS). The cells tend to undergo negative effects when the enhanced level of ROS persists over a long term. Secondly, the physical dimensions of NPs can cause some changes of cellular machinery and cytoskeleton network after their internalization into cells. Thirdly, the internalized NPs can interfere with intracellular signaling pathways and subsequently result in a cascade of side effects. Besides that, some degradation products of NPs, which cannot be easily discharged from cells, may also induce ROS significantly and affect cell homeostasis [10–12]. Furthermore, small NPs may result in very slow clearance* in vivo* that their potential deleterious effect could persist for long period [13, 14]. When NPs can be applied on stem cells-based cardiac repair, cautious and systematic assessment of biosafety risks is particularly important since stem cells are more fragile and particularly sensitive to toxicants than immortal cell lines [13].

## 3. Combination of Stem Cells and NPs

**Figure 1:**
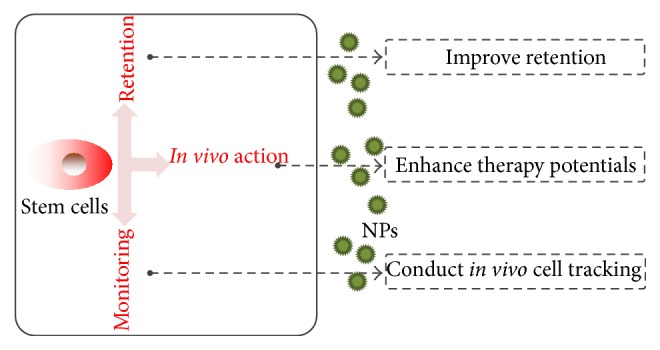
Schematic illustration of NPs-assisted stem cell therapy.

Multiple mechanisms, such as stimulation of angiogenesis and promotion of cardiomyocytes regeneration, have been involved synergistically in stem cell-based cardiac repair [15]. However, some barriers significantly limit their therapeutic effect in clinic trials. The first challenge facing stem cell therapy for cardiac repair is their low cell retention during and immediately after transplantation. Afterwards, their repair capacity and survival are obviously inhibited by the harsh ischemic microenvironment [16]. Besides that, it is still challenging to monitor the behaviors and fates of stem cells in myocardium [17, 18]. Recently, NPs have been considered as useful tools to counter these drawbacks (Figure 1). These nanostructured vehicles, loaded with functional agents, can be easily internalized into stem cells to realize efficient gene engineering, cell labeling, and retention enhancement. In this context, stem cells can be potentially enhanced for cardiac repair.

## 4. NPs for Gene Engineering in Stem Cells

In animal research, genetic engineering has been widely adopted in stem cells to enhance their paracrine secretion and survival* in vivo*, which can subsequently improve angiogenesis, relieve ventricular remodeling, and enhance global heart function [19–21]. Various therapeutic genes, such as proangiogenic and antiapoptotic genes, have been delivered through gene vectors for establishing genetically engineered stem cells for cardiac repair [22–24]. To this end, continuous effort has been made towards the development of effective and biocompatible gene vectors. Unfortunately, it is relatively difficult to transfect the primary cultured stem cells without impacting their characteristic of “stemness” and cell viability [25, 26]. Traditional viral vectors usually allow efficient gene delivery and stable gene expression in the previous studies. However, their applications in clinic are currently limited due to the potential oncogenic transformation, immune responses, and limited gene-loading volume [27, 28]. It is of great demand to develop novel nonviral gene vectors to establish genetically engineered stem cells for* in vivo* cardiac repair.

In the last decade, diverse types of NPs have been designed and synthesized elaborately as nanostructured vehicles to deliver therapeutic genes into somatic cells [7, 29–31]. NPs-based establishment of genetically engineered stem cells has also been investigated as a promising interdisciplinary strategy for tissue repair [26, 32–34]. Compared with viral vectors, NPs show their biocompatibility in cells and tissues. With extensive effort being made to elicit higher gene delivery efficacy, NPs-based vectors may be superior to viral analogues in future clinical trials.

### 4.1. Types of NPs-Based Gene Vector

Liposome is a spherical particle consisting of a lamellar phase lipid bilayer and an aqueous inner cavity. Liposomes with mean diameter of 100 nm can be classified as NPs and used for delivering genes into stem cells. Therapeutic genes (DNA/RNA) can be encapsulated into the internal aqueous phase of liposomes or bound onto their surface. The liposome/gene complexes, which are known as “lipoplexes,” can protect genes from degradation and nonspecific binding during transfection process [35]. Several commercially available and artificial liposomes have been used for delivering genes into stem cells for cardiac repair [35, 36]. Also, they have been used as references in gene transfer studies to evaluate the performance of new gene vehicles [37–39]. Even if liposomes were among the earliest vehicles for genes delivery into animal cells, they exhibited relatively low efficiency in primary stem cells. One report even claimed liposomes were unable to transfect human mesenchymal stem cells (MSCs) [25]. Also, due to their interaction with cell membrane, liposomes exhibit high cytotoxicity, which may injure fragile stem cells during genetic engineering and accelerate cell apoptosis in ischemic microenvironment [40].

Polymers, which range from natural to synthetic, can be generated via polymerization of monomers [41]. Over the last decade, many kinds of polymer-based NPs, such as dendrimers, polyethylenimine (PEI), and chitosan, have been developed and applied as gene vectors. Negatively charged genes can interact with their high densities of positively charged groups, most often primary amines, to form the condensed “polyplexes” [42]. Polyplexes are normally positively charged particles that can be bound to the anionic sites on cell membrane and subsequently internalized by cells. Cationic polymers can protect genes from degradation and facilitate their escape from endosomes and lysosomes. Importantly, polymers can be easily surface-modified to improve their transgene performance, such as increasing efficiency, reducing cytotoxicity, and realizing specific targeting [42]. For example, our group modified poly(amidoamine) nanoparticles with arginine to promote cell membrane penetration. With the double positively charged arginine residues, siRNA of prolyl hydroxylase domain protein 2 could be delivered efficiently and significantly enhance the survival of grafted MSCs in ischemic myocardium [43]. Recently, nanogels, which are crosslinked spherical hydrogel with nanosize, have been developed as a novel type of polymer-based vector and may be applicable for gene engineering in stem cells [44].

Inorganic NPs have emerged recently as a novel and attractive type of gene vector [45]. They can be used alone or blended with organic materials to conduct cellular gene transfer, since they can load genes* via* absorption or conjugation and then be internalized by the cells. Up to now, several types of inorganic NPs, including calcium phosphate, magnetic nanobeads, carbon nanotubes, silica, gold, and quantum dots, have been developed for gene delivery in stem cells [25, 46–51]. Although inorganic NPs show relatively moderate transfection efficiencies in most cell lineages, they possess their own advantages of simple fabrication and low cytotoxicity [45].

Each type of NPs vector has been widely used for gene engineering in stem cells. And the blended gene vectors, which integrate multitypes of materials (lipid, polymer, peptide, inorganics, etc.) into one platform, have been designed for higher transfection efficiency and biocompatibility (Table 1). For example, Song et al. developed a family of serum-resistant cationic lipids (lysinylated, histidylated, and arginylated cholesterol)-coated PEI to condense DNA as “lipopolyplexes,” which simultaneously improved transfection efficiency and reduced cytotoxicity in bone marrow stem cells [51]. Recently, Muroski et al. reported Bax inhibiting peptide-modified gold NPs as a good candidate for gene engineering in MSCs. The study confirmed that transfection efficiency achieved 80% and the overexpression of the desired protein lasted for 4 days. Besides that, this strategy exhibited no obviously negative impact on cell viability (93.8%) and surface markers (CD-90, CD-54, and CD-45) of MSCs [26].

### 4.2. Mechanisms of NPs-Based Gene Transfer

**Figure 2:**
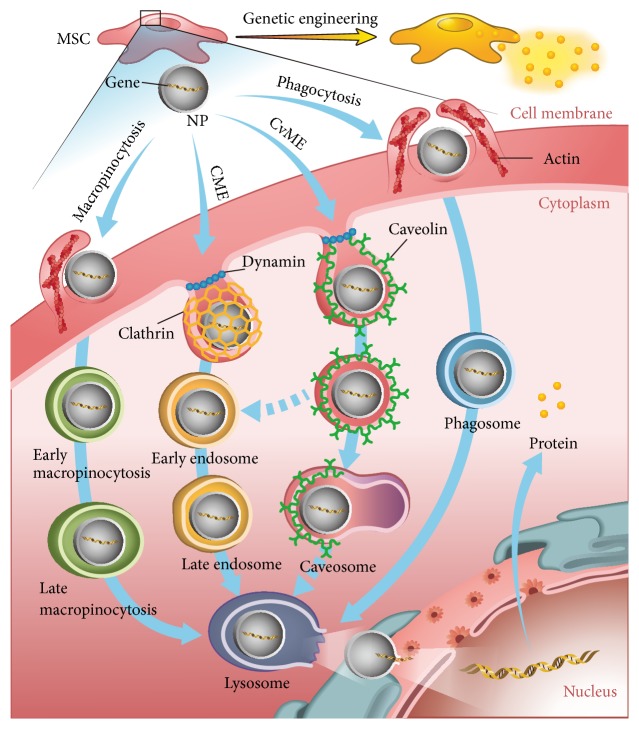
Schematic illustrations of potential mechanisms of NPs-based endocytosis while delivering therapeutic gene into MSCs.

The comprehensive understanding on the mechanisms of NPs-based gene delivery is necessary for the rational design of NPs vectors. The main mechanism is known as endocytosis pathways, including clathrin-mediated endocytosis (CME), caveolae-mediated endocytosis (CvME), macropinocytosis, and phagocytosis (Figure 2) [52]. Although endocytosis can occur in any type of stem/progenitor cells, it is still unclear whether all four pathways are involved in each type of NPs [52, 53].

After internalization, most of NPs gene complexes tend to be fused into endosomes and lysosomes and eventually escape from them [52]. Cationic NPs vectors are capable of escaping from the endolysosomes easily through their “proton sponge” effects. In the endolysosomes, the protonated nitrogen atoms of cationic NPs can consume endosomal protons and subsequently increase endosomal chloride anion, which enhance the inner osmotic pressure swells and rupture the endolysosomes. As a result, the complexes escape and transport to the appropriate sites where they can exert their functions [52, 54]. Besides that, lipoplexes may conduct another strategy, known as flip-flop mechanism, to escape from endolysosomes. The cationic structure of lipoplexes can interact with anionic monolayer lipid from cytoplasmic leaflet of endolysosomes membrane and then release genes directly into cytoplasm [55].

It has been known that some factors, such as cell situation, transfer duration, transfection temperature, and weight ratios of NPs to gene, contribute to the resultant efficiencies of NPs-based gene transfer in stem cells [52]. Therefore, transfection protocol of NPs has to be optimized over and over again before they can be used to establish genetically engineered stem cells. Moreover, long-termed and stable expression of therapeutic genes in stem cells is essential for efficient cardiac repair* in vivo* [56]. Hence, controlled release of genes needs to be elaborated through diverse modification on NPs.

## 5. NPs for Stem Cell Tracking

**Figure 3:**
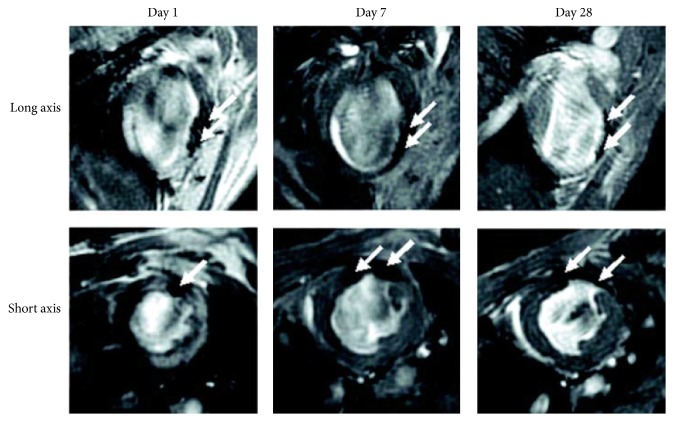
*In vivo* magnetic resonance images of mouse hearts injected with superparamagnetic NPs-loaded, cardiac-differentiated mouse embryonic stem cells. Scans were performed at 1, 7, and 28 days after cell transplantation in both the long-axis and short-axis orientations. NPs-loaded cells were shown as dark regions (white arrows) in the left ventricular wall in or near the infarct zone. Reprinted with permission from Ebert et al. [61].

After transplantation, stem cells reside and play a role in the microenvironment of ischemic myocardium. However, comprehensive understanding of* in vivo* behaviors of stem cells is still lacking, which results in our confusion of the contradictory results from current clinical trials [62–64]. Hence, it is of great demand to evaluate the survival, migration, and differentiation of transplanted stem cells in myocardium and underlying mechanisms behind these behaviors. To achieve this end, indirect and direct labeling techniques on stem cells have been developed in last decade. For indirect labeling approach, reporter genes could be transfected and overexpressed in stem cells. Direct labeling approach, by contrast, can be achieved easily by incubating stem cells with labeling agents [65]. As direct labeling agent, NPs display powerful superiority with their biocompatibility, real-time detection, and capability of functional modification [66]. Therefore, NPs have the potential as labeling agent to track the transplanted stem cells in myocardium. And endocytosis mechanisms of NPs labeling agents could be the same as those of NPs gene vectors. NPs labeling agents include magnetic and optical properties and can be* ex vivo* detected directly. Comparatively, magnetic NPs have been widely utilized as a stem cell labeling agent in cardiac repair because magnetic resonance imaging (MRI) can detect cell signals and meanwhile offer two- or three-dimensional imaging of cardiac tissue [67]. Magnetic NPs can change the relaxation rates of the water protons in nearby tissues, which make conspicuous images of NPs on post-contrast-enhanced MRI (Figure 3) [61].

### 5.1. MRI Tracking

Superparamagnetic iron oxide (SPIO) NPs can be observed as hypointensity on T2-relaxation MRI. SPIO NPs can label stem cells in myocardium without affecting cell proliferation, differentiation, migration, and viability [68, 69]. However, other studies demonstrated that MRI might overestimate survival rate of SPIO-labeled stem cells and could not track them for a long time, as macrophages within myocardium could phagocytose the discharged SPIO from the dead stem cells over time and result in false hypointensity on MRI [70, 71]. Whatever, SPIO still can be applied to guide and assess the transplantation of stem cells into targeted tissue area [69].

As paramagnetic probe, gadolinium (Gd) can generate hyperintensity on T1-weighted sequences and is among the most-widely used in MRI. Gd^3+^ ion usually forms a complex with the chelating ligand, such as diethylenetriaminepentaacetic acid (Gd-DTPA). However, Gd complexes have an inherently relatively low relaxation and cannot pass through cell membrane easily [60]. For the purpose of stem cell tracking, NPs can be used to facilitate cellular uptake and concentrate Gd in cytoplasm. For instance, small clusters of Gd^3+^ ions can be encapsulated by single-walled carbon nanotubes and internalized efficiently by MSCs [72]. Another report conjugated Gd with liposome NPs to generate Gd-liposome, which can label MSCs and be tracked* in vivo* for at least 20 days [73].

### 5.2. Optical Tracking

Some types ofNPs, such as silica NPs, gold nanorods, and carbon nanotubes, have the capacities to be conjugated with optical agents, which can be delivered into stem cell labeling and detected directly* ex vivo* [57, 74]. They provide a low-cost and effective approach and can be noninvasively tracked repeatedly. However, fluorescence can be absorbed and scattered, which leads to a limited penetration depth (<4 cm) from skin surface. This disadvantage restricts their application on small animals or superficial tissues in humans [58, 59]. Compared with the traditional optical labeling, several novel types of optical NPs, including quantum dots and upconversion NPs, possess enhanced tissue penetration ability and sensitive detectability, which provide the potential for clinical application of stem cells tracking in human hearts in future [75, 76].

### 5.3. Multimodality Tracking

The ideal cell labeling agent should provide complementary information of* in vivo* cell behaviors with high sensitivity and resolution [64]. Unfortunately, no single modality can be sufficient to meet all needs for tracking (Table 2). The combination of multimodal agents, such as MRI contrast agents, optical agents, and radionuclide, can yield synergistic superiority over any single modality [65]. NPs possess large surface areas and can be functionally modified to incorporate multiple labeling agents [66, 77–79]. However, the elaborated hybrid properties on NPs need integration of nanotechnology, imaging, biology, and medicine. In future, NPs-based multimodal labeling agents could be a trend to evaluate behaviors of stem cells on anatomic and functional levels.

## 6. NPs for Stem Cell Retention

The poor stem cell retention after cell delivery in targeted myocardium is a major limitation to therapeutic efficacy. Majority of transplanted cells can be washed out from coronary blood flow or squeezed out along with myocardium contraction during and immediately after cell transplantation [80]. It is reported that the rapid loss rate of stem cells occurs during and immediately after transplantation, regardless of the cell source and transplantation route [81, 82]. Kang et al. reported that only 1.5% (range, 0.2%–3.3%) of transplanted stem cells accumulated in the myocardium at two hours after intracoronary infusion in patients with myocardial infarction [83].

Magnetic NPs have been applied to enhance cell retention in myocardium. In previous studies, stem cells were labeled with magnetic NPs and then transplanted into myocardium by intramyocardial injection, retrograde coronary venous, and intracoronary infusion. The magnets were placed 0~1 mm above the injured myocardium during and after cell transplantation. It demonstrated that the retention rate of magnetic NPs labeled stem cells could be significantly enhanced under the magnetic field [84–87]. Recently, Cheng et al. reported functionalized SPIO as novel tool to enhance cell retention. In their study, two types of antibodies, which could link the exogenous bone marrow-derived stem cells and endogenous CD34-positive stem cells to the injured cardiomyocytes, were bound onto SPIO successfully. Under the magnetic field, the intravenously infused SPIO accumulated in myocardium and subsequently concentrated the transplanted CD34 positive stem cells into the targeted ischemic myocardium [88].

However, it is unclear whether magnetic accumulation of stem cells could enhance the risk of microembolization in coronary when delivering stem cells through coronary infusion. One recent study compared different magnetic field intensity (0.15, 0.3, and 0.6 Teslas) from the magnets and found that high magnetic field might have no additional therapeutic benefits though it had the highest cell retention. High magnetic intensity may result in unfavorable microembolization and consequently undermine functional benefits of stem cell therapy [89]. Therefore, the magnetic field intensity and exposure time should be further optimized in future studies.

## 7. Conclusions and Outlook

Collectively, NPs-based approaches serve as attractive technologies to overcome significant challenges associated with stem cell-based cardiac repair. NPs can assist stem cells to achieve higher therapy potential. Even though various NPs have been developed in animal research, biosafety concerns are still the main challenge before they can be translated into clinic. Comprehensive understanding of the characteristics of NPs can benefit their biocompatible design or surface modification. Moreover, multimodal imaging is a trend for stem cells tracking in future. Various types of NPs can be the candidates to integrate multiple labeling properties into one particle. Besides that, it is also a task to enhance the resolution and sensitivity of NPs labeling probes in deep tissues, such as in heart. Besides that, NPs can be an excellent platform to integrate multiple applications together. In future, hybrid NPs could be developed to simultaneously deliver therapeutic genes, drugs, and labeling agents into stem cells, which could generate highly reinforced stem cells for cardiac repair and labeling. In addition, NPs with specific characteristics have been explored to modulate stem cell biology. Recent report demonstrated that incorporating electrically conductive silicon nanowire into neonatal and human induced pluripotent stem cells-derived cardiac spheroids could create electrically conducting microenvironments, which subsequently induced significantly more advanced cellular structural and contractile maturation [90]. More efforts are needed to explore more effect of NPs-based modulation on other types of stem cells. In addition, mechanisms of stem cell-based cardiac repair need deep exploration for further assistance from NPs. We believe that NPs can benefit for future biomedical research in a large-scale field with their nanoscale structure.

## Figures and Tables

**Table 1:** Examples of NPs-based gene delivery in stem cells.

Stem cells	Species	Cell source	Type of NPs	NPs vectors	Internalization	*In vivo* test	Disease model	References
MSCs	Mouse	Bone marrow	Polymer	Hyperbranched poly(amidoamine)	Not reported	Yes	MI	[32]
SkMs	Human	Skeletal muscle	Liposome	Cholesterol-DOTAP liposome	Not reported	Yes	MI	[31]
MSCs	Rat	Bone marrow	Inorganics	Calcium phosphate	Not reported	No	—	[41]
MSCs	Human	Bone marrow	Blended	PEI-coated multiple QD bundled NPs	96.71% of NPs internalization after 6 h (QD655)	No	—	[45]
MSCs	Rat	Bone marrow	Blended	Cationic lipids (lysinylated, histidylated, or arginylated cholesterol)-coated PEI	99.6% of NPs internalization after 4 h (lysinylated cholesterol-coated PEI)	No	—	[46]

MI, myocardial infarction; MSCs, mesenchymal stem cells; SkMs, skeletal myoblasts.

**Table 2:** Evaluation of NPs labeling agents for stem cells.

NPs labeling modality	Advantages	Disadvantages
MRI modality	High spatial resolution (25–100 *μ*m) [57, 58] Excellent tissue penetration depth (no limit) [58] Allowing quantitative measurements	Low sensitivity (mM to *μ*M) [57, 58] Long scan time (minutes to hours) [59] High cost

Optical modality	High sensitivity (nM to pM) [58]	High scattering High absorption in tissue Short penetration depth (<4 cm from skin surface) [60]
